# Evaluation of region selective bilirubin-induced brain damage as a basis for a pharmacological treatment

**DOI:** 10.1038/srep41032

**Published:** 2017-01-19

**Authors:** Matteo Dal Ben, Cristina Bottin, Fabrizio Zanconati, Claudio Tiribelli, Silvia Gazzin

**Affiliations:** 1 Italian Liver Foundation (Fondazione Italiana Fegato), AREA Science Park, Trieste, Italy; 2 Department of Medical Sciences (Dipartimento di Scienze Mediche), Ospedale di Cattinara, Univestità degli Studi di Trieste, Trieste, Italy

## Abstract

The neurologic manifestations of neonatal hyperbilirubinemia in the central nervous system (CNS) exhibit high variations in the severity and appearance of motor, auditory and cognitive symptoms, which is suggestive of a still unexplained selective topography of bilirubin-induced damage. By applying the organotypic brain culture (OBC: preserving *in vitro* the cellular complexity, connection and architecture of the *in vivo* brain) technique to study hyperbilirubinemia, we mapped the regional target of bilirubin-induced damage, demonstrated a multifactorial toxic action of bilirubin, and used this information to evaluate the efficacy of drugs applicable to newborns to protect the brain. OBCs from 8-day-old rat pups showed a 2–13 fold higher sensitivity to bilirubin damage than 2-day-old preparations. The hippocampus, inferior colliculus and cerebral cortex were the only brain regions affected, presenting a mixed inflammatory-oxidative mechanism. Glutamate excitotoxicity was appreciable in only the hippocampus and inferior colliculus. Single drug treatment (indomethacin, curcumin, MgCl_2_) significantly improved cell viability in all regions, while the combined (cocktail) administration of the three drugs almost completely prevented damage in the most affected area (hippocampus). Our data may supports an innovative (complementary to phototherapy) approach for directly protecting the newborn brain from bilirubin neurotoxicity.

Neonatal hyperbilirubinemia is a common and benign event in newborns, characterized by an increased level of unconjugated bilirubin (UCB), which has antioxidant effects^1^. The vast majority of UCB exists in the blood bound to its carrier protein albumin. However, a small fraction of UCB remains unbound as free bilirubin (Bf), which is responsible for the pathological effects on the central nervous system (CNS)^2^,^3^. When hyperbilirubinemia is left untreated, both bound and unbound forms of bilirubin are elevated, with the fraction of Bf increasing as the amount of available albumin decreases^4^,^5^. Presently, deaths due to hyperbilirubinemia are rare in Western countries thanks to the feasibility and efficacy of phototherapy. However, in past years, there has been a resurgence of kernicterus (the most severe and permanent form of bilirubin brain toxicity, RC0180; RP0060)^6^. If added to the still occurring severe damage and death in low and mild-incoming countries^6^, as well as the lifelong risk of developing kernicterus experienced by Crigler-Najjar Type I patients (OMIM218800; ORPHA79234; ICD-10: E80.5), the consequences of hyperbilirubinemia continue to merit attention, and it is crucial to improve the risk assessment and the therapies for this condition.

It is well accepted that the clinical symptoms of bilirubin toxicity in the brain reflect the selective topography of bilirubin-induced damage: motor disorders and athetosis (basal ganglia and cerebellum), auditory dysfunction (inferior colliculus), and learning impairments (hippocampus and cerebellum)^7^. Nevertheless, this pathological condition still has unexplained variability in the severity and occurrence of the above reported symptoms^8^. A possible reason for this variability has been attributed to the level and duration of hyperbilirubinemia^9^. As learned from other neonatal neurological diseases, alternative explanations exist. As described in Rice and Barone, windows of CNS vulnerability to stimuli have been documented to strongly depend upon the developmental events occurring at the time of exposure to a toxicant, rather than before or after, and might influence the outcome^10^.

To map bilirubin targets in the post-natal brain during development and to elucidate the mechanisms as a basis for possible therapeutic intervention, we used the organotypic brain culture (OBC) technique^11^ to study bilirubin neurotocicity. OBCs are slices of a specific region of the brain that conserve cellular heterogeneity and connections^12^ and exhibit synaptic plasticity and can reveal mechanisms of pathological insults comparable to what is obtained *in vivo*. OBCs are easily grown *in vitro* thus allowing for direct exposure to outside agents. They, therefore, represent an ideal tool to assess *ex vivo* the effect of a compound such as Bf on a specific CNS region^13^. In addition, OBCs can be prepared from animals at different postnatal ages, thus allowing one to mimic the various stages of CNS maturation. We also evaluated the use of various drugs aimed at directly protecting the brain pharmacologically, as an innovative treatment to be used as a complement to traditional phototherapy.

## Results

### Recovery of OBCs in Bf medium

**Figure 1:**
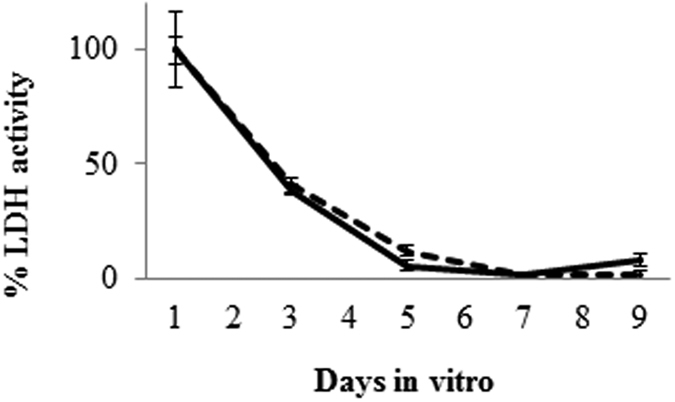
Representative picture of OBC recovery after slicing. Comparison of membrane damages (LDH release) in usual OBC medium (solid line) and in Bf-adapted medium (dotted line) after cutting (refer to culture medium paragraph in Material and Methods section). The results are expressed as the percentage *vs.* maximum release (day 1). Data are expressed as the mean ± SD of three repetitions.

To compare the viability of OBCs in standard medium *vs.* OBC media (see “Methods”) LDH release assay was performed. As shown in Fig. 1, the OBCs showed a significant LDH release immediately after slicing (which reflects procedural stress). This initial increase in LDH release decreased to normal levels in approximately 5 days (recovery time). No differences in LDH release were observed between OBCs cultured in standard *vs.* OBC media indicating that the modified media does not affect this assay. No differences were detected for any brain region or at any post-natal age (data not shown). Based on these results, Bf treatment was initiated at day 6 *in vitro* to allow for the complete recovery of the OBCs.

### Regional sensitivity to bilirubin toxicity

Three viability tests were used to extensively follow the damage induced by Bf treatment. Each monitored a different typology of damage: membrane leakage (LDH release assay), apoptosis induction (Hoechst staining) and impairment of mitochondrial activity (MTT test).

#### LDH release

**Figure 2:**
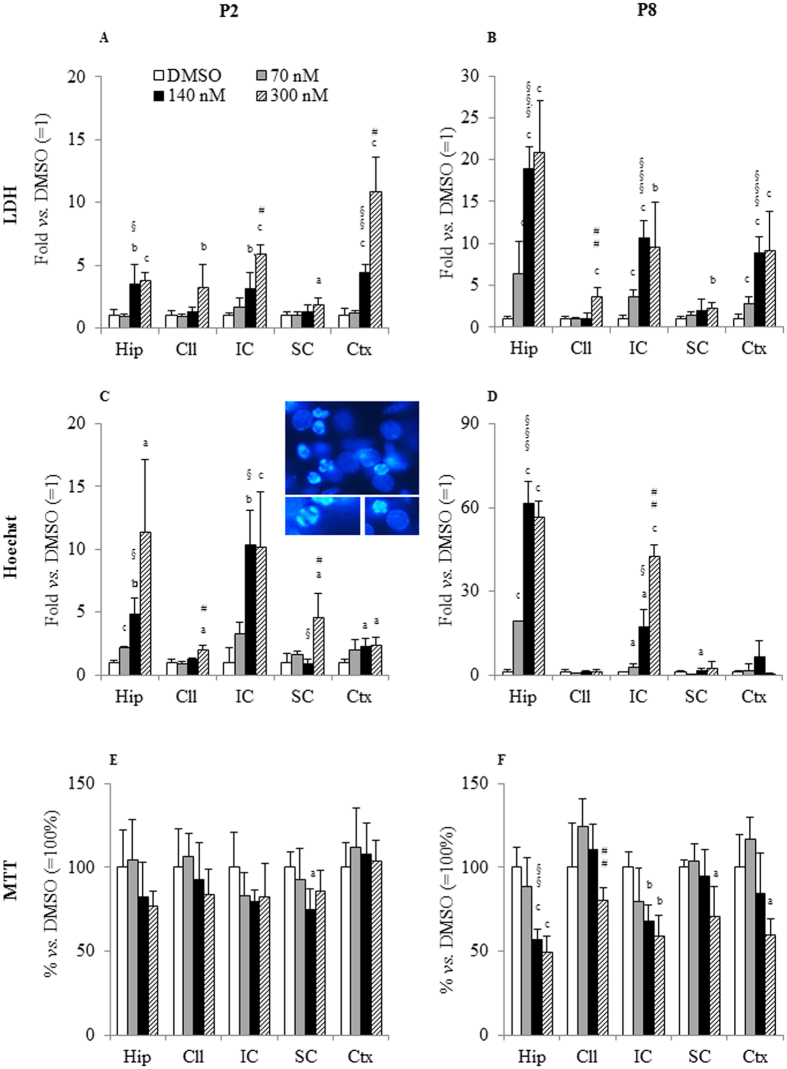
Results of viability tests of P2 and P8 OBCs challenged with 70, 140 and 300-nM Bf. (**A**) LDH release in P2 OBCs; (**B**) LDH release in P8 OBCs; (**C**) Hoechst staining of P2 OBCs; (**D**) Hoechst staining of P8 OBCs; (**E**) MTT assay of P2 OBCs; (**F**) MTT assay of P8 OBCs. LDH release and Hoechst are expressed as fold change of control (DMSO = 1). MTT is expressed as % change of control (DMSO = 100%). Data are expressed as the mean ± SD of three-five repetitions. Statistical significance: (a) p < 0.05, (b) p < 0.01, (c) p < 0.001 *vs.* DMSO; ^§^p < 0.05, ^§§^p < 0.01, ^§§§^p < 0.001 70-nM *vs.* 140-nM; ^#^p < 0.05, ^##^p < 0.01, ^###^p < 0.001 140-nM *vs.* 300-nM. Hip: hippocampus; Cll: cerebellum; IC: inferior colliculus; SC: superior colliculus; Ctx: cortex.

OBCs obtained from 2-day-old rat pups (Fig. 2A) did not show statistically significant increases in LDH activity at 70-nM Bf (sensitivity threshold, see “Methods” for details). Conversely, 140-nM Bf (toxic concentration) resulted in a toxic effect on the hippocampus (Hip: 3.5-fold *vs.* DMSO, p < 0.01), inferior collicula (IC: 3.2-fold *vs.* DMSO, p < 0.01) and cerebral cortex (Ctx: 4.4-fold *vs.* DMSO, p < 0.001). When Bf was increased to 300-nM (higher level to exacerbate the damage), a dose-dependent effect was observed in the IC and Ctx (2.0- and 2.5-fold, respectively, *vs.* OBCs treated with 140-nM Bf, p < 0.05; and 5.9-fold and 10.8-fold, respectively, *vs* OBCs treated with DMSO, p < 0.001), with the latter showing the maximum damage. No dose effect was observed in the Hip (3.8-fold *vs.* DMSO, p < 0.001). Only at 300-nM Bf concentration was a loss of membrane integrity observed in the cerebellum (Cll: 3.3-fold, p < 0.01) and superior collicula (SC: 1.8-fold, p < 0.05).

In OBCs from 8-day-old animals (Fig. 2B), irrespective of the bilirubin concentration, the Hip displayed the maximum toxicity, followed by the IC and Ctx. In these three regions, LDH activity was already significantly increased after 24 hrs at 70-nM Bf (6.4-, 3.6- and 2.8-fold, respectively, p < 0.001). Increasing bilirubin concentration (140-nM) increased the damage in the Hip, IC and Ctx (to 19.0-, 10.6- and 8.9-fold *vs.* DMSO, p < 0.001). On the contrary, no damage was detected in the Cll and SC at 140-nM Bf. At 300-nM Bf, we did not observe a further increase in LDH release in the Hip, IC and Ctx. As in the case of OBCs from 2-day-old animals, statistically significant changes were present in the Cll (3.6-fold, p < 0.001) and SC (1.8-fold, p < 0.01) after only 24 hrs of exposure at 300-nM Bf.

#### Hoechst staining

The numerical data and a representative picture of the chromatin condensation are shown in Fig. 2C and D. In OBCs from 2-day-old rats (Fig. 2C), 70-nM Bf treatment resulted in statistically significant apoptosis in only the Hip (2.2-fold *vs.* control, p < 0.001), with a clear dose effect when increasing Bf to 140-nM (2.3-fold *vs.* OBCs treated with 70, p < 0.05; p < 0.01 *vs.* OBCs treated with DMSO) and 300-nM Bf (12.0-fold *vs.* OBCs treated with DMSO, p < 0.001). At 140-nM Bf, apoptosis was significant in the Ctx (2.2-fold, p < 0.05), without any further increase at 300-nM, and in the IC (10.4-fold, p < 0.01 *vs.* control, 3.1-fold *vs.* OBCs treated with 70-nM Bf, p < 0.05), again with no change when increasing concentration to 300-nM Bf. Moreover, 300-nM Bf was needed to induce apoptosis in the Cll (2.0-fold *vs.* control, p < 0.05) and SC (4.5-fold *vs.* control, p < 0.05).

Hippocampal slices from more developed animals (P8 – Fig. 2D) confirmed a high sensitivity to the low bilirubin dose of 70-nM (p < 0.001), with further increases at both 140 and 300-nM (61-fold *vs.* control, p < 0.001, and 3.7-fold *vs.* OBCs treated with 70-nM Bf, p < 0.01). The IC was also damaged after each treatment, but with a clear dose-dependent effect (2.9-fold at 70-nM, p < 0.01; 17-fold at 140-nM, p < 0.01; and 43-fold *vs.* OBCs treated with DMSO, p < 0.001). No significant bilirubin-induced apoptosis was observed in the Cll or Ctx.

#### Mitochondrial activity

Despite some change in MTT activity (Fig. 2E), no significant changes were found in P2 OBCs exposed to all Bf concentrations, except for a 25% decrease (p < 0.05) in the SC exposed to 140-nM Bf.

In P8 OBCs (Fig. 2F), a significant reduction in mitochondrial metabolism was detected in the Hip (p < 0.001) and IC (p < 0.01) exposed to Bf concentrations of 140 and 300-nM. The Ctx and SC showed a significant reduction in mitochondrial activity only when exposed to the maximal dose (300-nM Bf, both p < 0.05). The Cll was insensitive to all Bf concentrations.

### Developmental sensitivity to bilirubin toxicity

Because brain exhibits significant post-natal development^10^, two different post-natal ages were compared. To highlight the developmental sensitivity to bilirubin toxicity, viability was expressed as a ratio of P8/P2 results (Table 1). A ratio higher than 1-fold indicates an increased developmental sensitivity in the LDH assay and Hoechst staining results; the opposite is true for MTT activity.

#### Developmental sensitivity to membrane leakage

LDH revealed a clear age-related increase in the sensitivity to bilirubin in the Hip when exposed to 140 and 300-nM Bf (P8 higher than P2) (both p < 0.001) (Table 1). A similar response was observed in the IC (at 140-nM Bf concentration, p < 0.01). No developmental sensitivity to bilirubin toxicity was observed in the Cll, SC or Ctx.

#### Developmental sensitivity to apoptosis

As shown in Table 1, at all bilirubin concentrations, Hip sensitivity was increased in P8 *vs.* P2 OBCs (p < 0.01 at 70-nM Bf and p < 0.001 at 140 and 300-nM Bf), while in the IC, an increase in apoptosis was observed at only the highest concentration (p < 0.001). No developmental difference was observed in the Cll, SC or Ctx.

#### Developmental sensitivity to in mitochondrial activity

Despite a trend of decrease in the Hip, IC and Ctx, no significant differences in the mitochondrial activity in these regions were observed (Table 1, lower panel).

### Screening of pathways of damage

Different analyses (histological, genomic, and enzymatic) were performed in OBCs treated with bilirubin to screen for biomolecular mechanisms of damage. Screening was performed at the post-natal age showing the maximal sensitivity (P8) and at the toxic Bf concentration of 140 nM for 24 hrs.

#### Hematoxylin and Eosin

**Figure 3:**
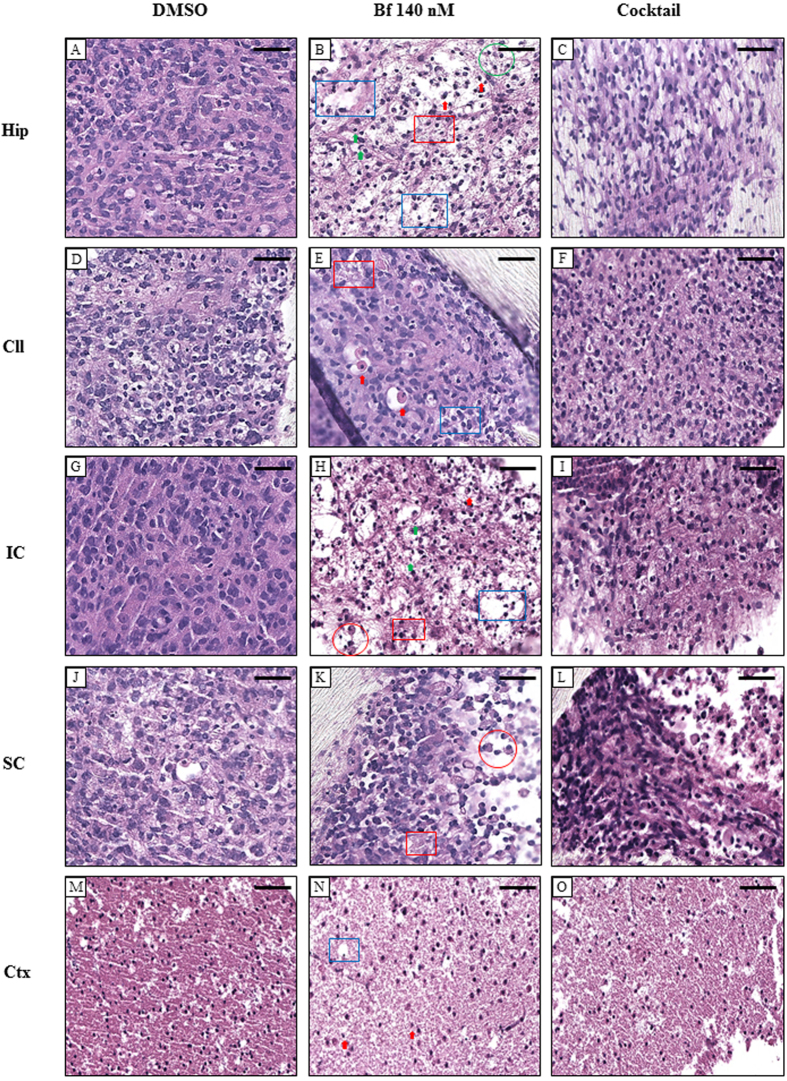
Histological analyses of the recovery of OBCs challenged with 140-nM Bf after treatment with cocktail. Images of H&E staining of OBCs treated with DMSO (**A,D,G,J,M**), 140 nM Bf (**B,E,H,K,N**) or cocktail (**C,F,I,L,O**) of Hip (**A,B,C**), Cll (**D,E,F**), IC (**G,H,I**), SC (**J,K,L**) and Ctx (**M,N,O**). Red arrow: apoptosis. Green arrow: microgliosis. Red circle: foam cells. Red square: fibrosis. Green circle: inflammation. Blue square: edema. Hip: hippocampus; Cll: cerebellum; IC: inferior colliculus; SC: superior colliculus; Ctx: cortex. Scale Bar 50 μm.

The damage to the Hip observed in the previously described viability tests was confirmed by histological evaluation. Tissue damage was evidenced by the appearance of large intracellular spaces, neuronal cell loss, apoptosis and some degree of necrosis. Edema, fibrosis, the presence of foam cells and microgliosis were also observed (see Fig. 3B = bilirubin treatment *vs.* A = control). Similar to the Hip, but to a lesser extent, the IC showed large intracellular spaces, apoptotic bodies and foam cells (Fig. 3H). Cellular death was also present in the Ctx (decreased cellular density, with a generalized weak nuclear staining – Fig. 3N). Some degree of degeneration was also present in the Cll, with peri-cellular edema, necrosis, and a slight detectable increase in apoptosis (Fig. 3E). The SC was only marginally affected by the exposure to bilirubin, with a negligible increase of cells with vacuoles and fibrillary matrix components with cellular debris, with respect to DMSO-exposed organotypic SC cultures (Fig. 3K).

#### Quantitative real-time PCR analysis of markers of damage

To evaluate the possible pathway of damage triggered by bilirubin in each region, we assessed the expression of selected genes as markers of specific biomolecular processes of damage (Table 2).

Oxidative imbalance was revealed in all regions with the exception of the Ctx. A 2.5- to 4-fold increase in expression of the inducible heme oxygenase-1 (*HO-1*) (a marker of oxidative stress) was detected in the Hip, IC (both p < 0.001), Cll (p < 0.01) and SC (p < 0.05). Sulfiredoxin 1 (*Srnx1*, involved in antioxidant metabolism, and induced in response to an oxidant milieu) was maximally modulated in the Hip and IC (p < 0.001 and p < 0.05, respectively), followed by the SC and Cll (p < 0.01 and p < 0.05, respectively).

The activation of the inflammatory pathway was assessed by the expression of multiple cytokines. Tumor necrosis factor alfa (*Tnfα*) mRNA was significantly induced in only the Hip and SC (p < 0.05 and p < 0.001, respectively). Interleukin 1β (*Il1β*) was maximally altered in the IC (p < 0.001), followed by the Hip (p < 0.01), Cll and SC (both p < 0.05).

The most striking alteration involved the interleukin 6 (*Il6*) and the cyclooxygenase (*Cox2*) genes, which were up-regulated approximately 300- and 50-fold, respectively, in the Hip and 100- and 60-fold, respectively, in the IC (all p < 0.001). In the third most damaged area (Ctx), *Il6* expression was up-regulated approximately 12.4-fold (p < 0.001), with *Cox2* expression up-regulated approximately 3.5-fold (p < 0.05). Both genes were modulated in the Cll (both p < 0.001) and in the SC (both p < 0.05).

#### Glutamate release in culture medium

The involvement of the glutamate excitotoxicity pathway was assessed by the quantification of glutamate release into the culture media (Table 2). Significant release was detected in the Hip, 2.4-fold (p < 0.001), and IC, 2.0-fold (p < 0.01), after bilirubin treatment.

### Evaluation of possible therapeutic approaches

**Figure 4:**
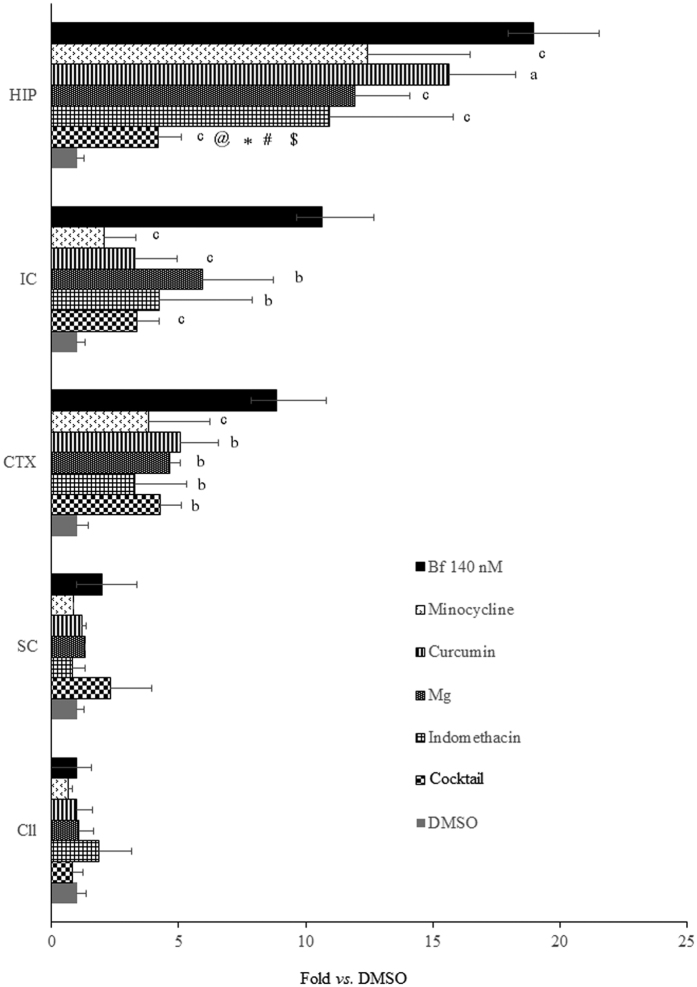
Viability improvement following treatment with bilirubin and therapeutic agents. Recovery from damage (LDH release) in challenged OBCs is expressed as fold change of control (DMSO = 1). Data are expressed as the mean ± SD of three-five repetitions. Statistical significance: (**a**) p < 0.05; (**b**) p < 0.01; (**c**) p < 0.001. @: p < 0.05 cocktail *vs.* indomethacin. *p < 0.01 cocktail *vs.* minocycline; ^#,$^p < 0.001 cocktail *vs.* curcumin and Mg, respectively. OBCs exposed to drugs alone as well as undamaged regions (Cll, SC) exposed to cocktail did not display signs of toxicity. Hip: hippocampus; Cll: cerebellum; IC: inferior colliculus; SC: superior colliculus; Ctx: cortex.

Finally, we used the OBCs as a screening platform to identify neuroprotective therapeutics. The LDH test was chosen to evaluate the protective effect of each treatment because of the level of sensitivity to Bf-induced damage that this test exhibited (Fig. 4). The main rationale for the drugs selected in this work was based on their potential to be used safely as new therapeutic treatments in severely jaundiced newborns^14^; therefore, only drugs already in clinical use, and known to cross the blood brain barrier were considered. The maximal dose not inducing adverse effects was experimentally identified, starting from the information available in literature for each compound. The current gold-standard drug treatment for bilirubin-based neurotoxicity, minocycline^15^,^16^, has been shown to suppress cerebellar hypoplasia in the Gunn rat^17^, the animal model for Crigler-Najjar type I and kernicterus. Minocycline was also effective in restoring Bf-induced damage in OBCs (Hip −35%, IC −80% and Ctx −57%, all p < 0.001) (Fig. 4). Curcumin, an antioxidant nutraceutical^18^,^19^, improved the viability of the challenged OBCs by approximately 69% (p < 0.001) in the IC, 43% (p < 0.01) in the Ctx and 18% (p < 0.05) in the Hip. MgCl_2_, a glutamate receptor blocker^20^,^21^, effectively improved viability by approximately 47% (p < 0.01), 44% (p < 0.01) and 37% (p < 0.001) in the Ctx, IC and Hip, respectively. Indomethacin, an anti-inflammatory drug^22^,^23^, increased the viability of OBCs by 63% (p < 0.01), 60% (p < 0.001) and 42% (p < 0.001) in the Ctx, IC and Hip, respectively. The cocktail of the 3 compounds greatly improved viability in the Hip (78%, p < 0.001 *vs.* Bf), while no additional difference in respect to single drugs was observed in the Ctx or IC (62%, 68%; both p < 0.001 *vs.* Bf). Exposure to a single drug or to the cocktail for 24 hrs did not increase LDH release in the OBCs compared to the DMSO control, indicating the safety of the dosage used in our system.

### Assessment of the damage improvements after treatment

As a final demonstration of drug efficacy, both the histological examination (Fig. 3C,F,I,L,O) and quantification of the markers of biomolecular pathways of damage were performed using the cocktail as stated above (Table 3).

In agreement with the viability improvement evidenced by the LDH release results, the cocktail approach coincided with a significant recovery in all the 3 damaged regions (Hip, IC and Ctx; Fig. 3C,I and O, respectively). However, the treatment did not completely reverse the damage due to Bf, as there were still marginal signs of damage present. Histological evaluation of Bf-induced damage in OBCs receiving the cocktail revealed a significant reduction in apoptosis and a moderate reduction of cellular edema in the Hip. The IC showed a reduction of edema with a conservation of cellular elements, while the Ctx had a reduction of necrosis and of the fibrotic scenario. The SC and Cll images showed slight histologic improvement (Fig. 3).

The observed improvement in the viability was confirmed at the molecular level by real-time PCR. The mRNA expression of markers for oxidative imbalance was slightly increased for *HO-1* in the Hip (increase of approximately 164% *vs.* bilirubin-treated OBCs, p < 0.01) (Table 3). The other regions did not show a significant change in *HO-1* expression after treatment with Bf and cocktail.

Treatment with the Bf + cocktail also showed a reduction of interleukin 6 (*Il6)* and cyclooxygenase 2 (*Cox2)* levels compared to slices exposed to Bf alone, thus indicating that the neuro-inflammatory markers were modulated by bilirubin treatment (Table 3) and the effect attenuated by the drugs. *Cox2* levels decreased by approximately 97% in the Hip, IC and Cll (p < 0.05, p < 0.01 and p < 0.01, respectively). *Il6* mRNA was reduced by approximately 80–90% in the Hip, IC, Ctx and Cll (all p < 0.01). Similar down-regulation (about of 95% *vs.* bilirubin-treated OBCs) compared to normal expression was also observed for *Il1β* in the Hip (p < 0.05), IC and Cll (both p < 0.01). Less impressive was the reduction of *Tnfα* expression, which was statistically significant in only the SC (p < 0.001).

In accordance with our previous results showing significant glutamate-mediated damage in only the Hip and IC, the cocktail significantly reduced glutamate release in these same regions (Hip p < 0.01 and IC p < 0.05).

## Discussion

Our data highlight a clear region-specific and developmental regulated in the brain’s sensitivity to bilirubin toxicity. The Hip emerged as the most sensitive brain region, followed by the IC. These data are in agreement with the most common clinical manifestations of bilirubin neurotoxicity: alterations in the auditory-evoked potentials^24^,^25^ and memory/learning deficits^26^,^27^. Additionally, our findings are in agreement with clinical findings^8^ that indicate damage to the IC and Hip^8^.

Despite being considered as a region resistant to bilirubin toxicity^28^, some evidence of bilirubin toxicity in the cerebral Ctx has been reported. Newborn electroencephalographic measurements have demonstrated that hyperbilirubinemia affects cerebro-cortical electrical activity in a time-limited manner^29^, and autopsy findings have shown apoptosis and necrosis in this area^30^. Moreover, nearly all *in vitro* evidence of the mechanisms of bilirubin-induced cytotoxicity have been obtained using primary cultures of neurons and astrocytes isolated from the cerebral Ctx^31^,^32^. In animal models, cortical sensitivity to bilirubin has also been shown by inhibiting the phosphorylation of synapsin I^26^, by observing histological abnormalities in the occipital cortex, and altering the balance of excitatory and inhibitory neurotransmitters^33^. Collectively, these findings suggest a possible cortical sensitivity to bilirubin. Though this sensitivity is probably less severe than that observed in other regions, potential damage to the Ctx likely needs to be considered in infants where bilirubin neurotoxicity is suspected.

Only P8 OBCs were sensitive to a bilirubin concentration of 70-nM (Bf) (Fig. 2), which is considered to be either not toxic or at least at the threshold of potentially causing damage^3^. This observation suggests an “age-dependent” sensitivity to bilirubin toxicity. This is in line with what has been reported in animal models for hyperbilirubinemia (Gunn rat), where the window of vulnerability to bilirubin toxicity has been observed at P6–10, a period preceded and followed by a much lower CNS vulnerability^34^,^35^,^36^. Unfortunately, no detailed biomolecular data are available on postnatal brain development in humans. Conversely, in rodents, the presence of a “dormant period” in the first 3 postnatal days has been described^37^. Thereafter, a sustained increase of NMDA receptor subunits^38^ and sensitivity to inflammation^39^ are observed in the second postnatal week. Along with these changes, the activation of neurogenesis, proliferation of non-neuronal cells, and increased apoptosis and remodeling in physiological tissues^37^ are all potential mechanisms for bilirubin toxicity^10^, contributing to the final toxic phenotype. This is in full agreement with the concept of the vulnerability of the developing brain to a toxic substance: “*if exposure occurs before or after an organ develops, it is less vulnerable to perturbation than if exposure occurs during development of that organ*”^10^, with respect to different brain insults and with our hypothesis that CNS sensitivity to UCB might be developmentally regulated.

Also of interest is the insensitivity of the Cll OBCs to acute (24 hrs) bilirubin insult. Cerebellar hypoplasia due to bilirubin toxicity is a landmark finding of animal models of hyperbilirubinemia^34^,^36^,^40^,^41^,^42^,^43^,^44^ and is reported in pre-term babies^45^,^46^. In our model, we found regions of the brain susceptible to an acute exposure (24 hrs) to UCB (Hip, IC) and others that are not. Specifically, for the Cll, our results suggest that a longer (chronic) exposure to toxic levels of UCB may be required to initiate the damage in this CNS region. This finding is in line with what has been reported in animal models. In both Gunn rats and UGT−/− mice, cerebellar damage took at least 7–9 days to become significant and was not detectable at early post-natal ages, despite the presence of hyperbilirubinemia early after birth^34^,^36^,^40^,^41^,^42^,^43^,^44^. Also in line with this hypothesis is the observation that 4 days of exposure to 140-nM Bf increased LDH release (>2.0-fold) in our Cll OBCs (data not shown).

In this study, we discovered a multifactorial etiology of bilirubin toxicity by cross-validating the data obtained from the analysis of mechanisms of damage (Table 3 and Fig. 3) and treatments (Fig. 4). Neuroinflammation (revealed by cytokine modulation – Table 3 and microgliosis – Fig. 3) and oxidative stress (*Ho1/Srnx1* modulation – Table 3, and foam cells observed in Fig. 3) were observed in all three damaged areas (Hip, IC and Ctx), with glutamate release present in only the Hip and IC OBCs (Fig. 3). Additionally, our findings regarding the neurological damage in Hip, IC and Ctx are in agreement with the histological findings at autopsy^30^,^45^. A part being in agreement with the literature based on cell lines and the animal model that we used as reference (see M&M), the pro-inflammatory action of bilirubin we observed well agreed with the two unique previous studies using cerebellar and hippocampal OBCs^40^,^47^. The discrepancy in the glutamate finding (not investigated in the Hip^47^, showing a temporary significant release in Cll OBCs exposed to UCB^40^, but never significant in the Cll in our acute scheme) might be explained by the different experimental set-ups, and with the amount of Bf used (20-nM in Barateiro *et al*.^40^ *vs.* 140-nM in. our work) being the major difference.

In less damaged regions, single drugs were effective in reducing tissue damage. Curcumin and indomethacin emerged as the most effective drugs in ameliorating the damage in the IC with similar results for indomethacin in the Ctx. In these regions, all therapeutic schemes applied matched with the protection offered by minocycline (Fig. 4), a drug shown to be fully effective in *in vivo* models of kernicterus^15^,^16^. Notably, in addition to the antioxidant and anti-inflammatory mechanisms, minocycline most likely acts as a chelator for bivalent ions, such as calcium which is probably strongly relevant in bilirubin encephalopathy, as demonstrated by Daood *et al*.^48^. However, minocycline use is not allowed in neonates due to its side effects, and safe calcium chelators are still not available in clinics. In agreement with the multifactorial pathway of damage, drugs applied as a cocktail seem to be to the most effective way to ameliorate the damage in the Hip, the most damaged region both in our experimental model and in newborns. Indeed, the additive effect of the cocktail *vs.* single drug administration (Fig. 4) indicates that bilirubin acts simultaneously on different, but not necessarily connected, pathways. Importantly, we observed no evidence of drug toxicity in damaged (Hip, IC and Ctx) or undamaged (Cll, SC) brain regions after exposure to either the single or cocktail drug treatments, indicating that the cocktail is potentially a more effective treatment to confer the maximum and widespread protection to the brain in severe hyperbilirubinemia.

In conclusion, the application of OBCs to study bilirubin neurotoxicity proved well-suited for the reproduction of findings observed *in vivo* and clinically. We have demonstrated a multifactorial toxic action of bilirubin, with some regional specificity, and we have shown *ex vivo* the efficacy of a new theoretical approach aimed at protecting the brain with drugs that are currently used in the clinical setting.

## Methods

### Organotypic brain culture preparation

Wistar Han^TM^ Rats, at 2 (P2) and 8 (P8) days after birth, were obtained from the animal facility of the University of Trieste. Animal experiments were performed according to the Italian Law (decree 87–848) and European Community directive (86-606-ECC). The study was approved by the animal care and use committee of the University of Trieste, along with regular communication with the Italian Ministry. Maximal effort was used to minimize the number of the animals used and their suffering, with the respect to the 3R rule. Immediately after sacrifice, the Hip, Cll, IC (all pathologically involved in kernicterus)^28^, SC and Ctx (considered resistant) were dissected and maintained in dissection medium (ice cold Gey’s Balanced Salt Solution plus D-Glucose 10 mg/mL) until use. A McIIwain tissue chopper (Gomshall Surrey, U.K.) was used to cut transversely 300 (SC, IC), 350 (Hip, Ctx) and 425 (Cll) μm slices. Healthy slices, selected for structural integrity under stereomicroscope inspection, were maintained in dissection medium for 60 min to allow clearing of cutting surfaces from preparation procedure stress. Slices were then transferred to sterile, semi-porous Millicell-CM inserts (PICM03050, Millipore, Darmstadt, Germany), fed by 1 mL of media and maintained at 37 °C, 5% CO_2_, 95% humidity in a humidified incubator^11^.

### Cultures medium

Standard culture medium (50% Basal Medium Eagle – BME - medium, 25% Hank’s Balanced Salt Solution - HBSS, 25% Heat-Inactivated Horse Serum, 1% L-Glutamine, 2% Penicillin/Streptomycin, 10 mg/mL glucose)^11^ was modified to allow for Bf quantification. Bf adapted medium (OBCs medium) was composed of 65% Basal Medium Eagle (Life Technologies Corporation, Grand Island, NY), 10% heat-inactivated Fetal Bovine Serum (Euroclone, Milan, Italy), 25% Hank’s Balanced Salt Solution (Sigma Aldrich, St. Louis, MO, USA), 1% L-Glutamine (Life Technologies Corporation, Grand Island, NY), 2% Penicillin/Streptomycin (Life Technologies Corporation, Grand Island, NY), 10 mg/mL D-Glucose (Sigma Aldrich, St. Louis, MO, USA). The media was changed the day after cutting and every two days thereafter. Slices were maintained in culture 5 days before starting Bf exposure, this amount of time was required to allow recovery from the stress of slicing (Fig. 1)^49^.

### Bf treatment

After 6 days *in vitro*, slices were challenged for 24 hrs with different bilirubin concentrations based on our experience *in vitro*^31^,^43^,^47^ and in the range of the *in vivo* brain Bf 50. The concentration of UCB dissolved in DMSO required to reach the desired Bf concentrations in the OBC medium was quantified according to Roca *et al*.^51^. Three concentrations of Bf were used: 70-nM (sensitivity threshold), 140-nM (toxic concentration), 300-nM (higher level to exacerbate the damage). Control slices were exposed to the same final concentration of DMSO used to dissolve the UCB.

### Viability tests

#### Lactate dehydrogenase release

The amount of total extracellular LDH in media, indicative of membrane leakage, was determined using a CytoTox-ONE™ Homogeneous Membrane Integrity Assay (G7891, Promega, Madison, WI, USA). After Bf exposure (24 hrs), the supernatant was collected, and the reaction was carried out according to the manufacturer’s instructions. The fluorescence (560_Ex_/590_Em_) was determined using an EnSpire Multimode Plate Reader (PerkinElmer, Waltham, MA, USA), and the background fluorescence subtracted. The amount of LDH released in treated slices was expressed as the fold change compared to the control slices.

#### Hoechst staining

Evidence of chromatin condensation, a marker of cell death by apoptosis, was obtained by administration of 1 μg/mL Hoechst 33258 (Sigma Aldrich, St. Louis, MO, USA). Slices treated with Bf or DMSO alone were fixed for 30 min at room temperature in 3% paraformaldehyde (PFA)^13^. Apoptotic cells were counted at 40X magnification, by fluorescence microscopy using a Leica DM2000 (Leica Mycrosystems Srl, Solms, Germany) by three separate individuals. At least three different fields were analyzed in each repetition. The results were expressed as the percentage of apoptotic cells relative to the total number (apoptotic plus unaffected) of cells in the control (100%).

#### Mitochondrial activity

Mitochondrial metabolic activity was assessed using a 1-(4,5-dimethylthiazol-2-yl)-3, 5-diphenylformazan (MTT) assay (Sigma-Aldrich, St. Louis, MO, USA). Post-challenge experiments, slices for each repetition were incubated with 0.5 mg/mL of MTT in media at 37 °C for 1 hr, harvested, and the precipitated salt dissolved in DMSO. Absorbance was detected at 562 nm using a LD 400C Luminescence Detector (Beckman Coulter, Milan, Italy). The results were expressed as the percentage of activity relative to controls (100%).

### Screening of pathway of damage

#### Histology

After Bf treatment, OBCs were immediately fixed in neutral buffered formalin and embedded in 4% paraffin. Tissue were sectioned to a thickness of 3 μm by a microtome (Microm-hm 340e- BioOptica, Milan, It), and dried in oven a 60 °C for one hour. Sections were stained with hematoxylin & eosin (H&E) using a Leica ST5020 Multistainer (Leica Microsystem, Milano, It). Histology was read by two independent pathologists, blinded to experimental design and treatment groups, at the Department of Pathology of Academic Medical Center Hospital of Cattinara. Images were collected by a D-Sight plus image digital microscope & scanner (Menarini Diagnostics, Firenze, Italy).

#### Quantitative real-time PCR of selected markers of bilirubin toxicity

Genes reported to be involved in the biomolecular pathways of neuronal damage were selected as markers of these pathway^47^,^49^,^52^ (see Table 4). The mRNA expression of the genes of interest was analyzed by quantitative real-time PCR. Total RNA was extracted using TRI Reagent^®^ RNA Isolation Reagent (Sigma-Aldrich, St. Louis, MO, USA), following the manufacturer’s instructions. Complementary DNA (cDNA) was synthesized with the High Capacity cDNA Reverse Transcription Kit (Applied Biosystems, Monza, Italy). For the quantitative real-time PCR, primers were designed using the Beacon designer 4.2 software (Premier Biosoft International, Palo Alto, CA, USA) on rat sequences available in GenBank (Table 1). The reaction was performed in a final volume of 15 μL in an iQ5 Bio-Rad Thermal cycler (BioRad Laboratories, Hercules, CA, USA). Briefly, 25 ng of cDNA and the corresponding gene-specific sense/antisense primers (250 nM each, with the exception of Cyclo-Oxygenase 2 -*Cox2* and Interleukin 1*β -Il1β*, 500 and 750 nM, respectively) were diluted in the Sso Advance SYBER green supermix (Bio-Rad Laboratories, Hercules, CA, USA). Amplification of target genes was accomplished using the following protocol: 3 min at 95 °C, 40 cycles at 95 °C for 20 sec, 60 °C for 20 sec, and 72 °C for 30 sec. The specificity of the amplification was verified by a melting-curve analysis, and non-specific products of PCR were not found in any case. The relative quantification was made using the iCycleriQ software, version 3.1 (Bio-Rad Laboratories, Hercules, CA, USA) by the ΔΔCt method, taking into account the efficiencies of the individual genes and normalizing the results to the housekeeping genes (Hypoxanthine guanine phosphoribosyl transferase: *Hprt*, Glyceraldehyde 3-phosphate dehydrogenase: *Gapdh*)^57^,^58^.

#### Glutamate quantification in culture media

The amount of extracellular glutamate (glu), a marker of bilirubin-induced excitotoxicity^31^,^59^,^60^, was quantified using a Glutamate Assay Kit (MAK004, Sigma-Aldrich, St. Louis, MO, USA). Briefly, after the Bf exposure, the supernatant was collected, and the assay was performed according to the manufacturer’s instructions. The absorbance (450 nm), proportional to the glutamate present, was determined using an EnSpire Multimode Plate Reader (PerkinElmer, Waltham, MA, USA). Glutamate release in media was expressed as fold change compared to the control slices.

### Screening of therapeutic drugs

Slices from P8 animals (the most sensible post-natal age), were exposed (for 24 hrs) to the following: (1) DMSO alone, as control; (2) 140 nM Bf–to induce the damage; (3) drugs alone–to assess possible toxic effects; (4) 140 nM Bf plus single drugs; and (5) 140 nM Bf plus the three principles simultaneously (cocktail). Drugs screened in this study included the following: curcumin, an antioxidant (50 μM, Sigma-Aldrich, St. Louis, MO, USA), magnesium chloride, a glutamate channel blocker, (10 mM, Sigma-Aldrich, St. Louis, MO, USA) as alternative to the bilirubin displacer magnesium sulfate^14^ already used in newborns, and indomethacin, an anti-inflammatory agent (50 μM, Liometacen, Promedica Srl. Parma, Italy). Minocycline was used at 60 μM (Sigma-Aldrich, St. Louis, MO, USA).

### Statistical analysis

Data were analyzed with GraphPad Prism version 5.00 for Windows (GraphPad Software, La Jolla California USA). Statistical significance was evaluated by paired two-tailed test. ANOVA was applied to the results in Table 2, where different post-natal ages have been compared. A p value < 0.05 was considered as statistically significant. The results are expressed as the mean ± SD of at least three independent repetitions.

## Additional Information

**How to cite this article:** Den Ben, M. *et al*. Evaluation of region selective bilirubin-induced brain damage as a basis for a pharmacological treatment. *Sci. Rep.* **7**, 41032; doi: 10.1038/srep41032 (2017).

**Publisher's note:** Springer Nature remains neutral with regard to jurisdictional claims in published maps and institutional affiliations.

## Figures and Tables

**Table 1:** Developmental sensitivity to bilirubin toxicity: the P8/P2 ratio of viability tests.

	Hip	Cll	IC	SC	Ctx
LDH					
70 nM	7.11	1.12	2.20	1.43	2.40
140 nM	5.44^c^	0.79	3.35^b^	1.51	2.03
300 nM	5.51^c^	1.12	1.61	1.22	0.85
Hoechst					
70 nM	8.95^b^	0.61	0.89	0.13	0.77
140 nM	12.70^c^	0.75	1.65	1.68	2.92
300 nM	4.97^c^	0.65	4.19^c^	0.51	0.12
MTT					
70 nM	0.85	1.17	0.96	1.12	1.04
140 nM	0.69	1.18	0.86	1.27	0.78
300 nM	0.64	0.95	0.71	0.83	0.57

Lactate dehydrogenase release (LDH - upper), Hoechst staining of apoptotic bodies (middle) and mitochondrial activity (MTT - lower) were expressed as the ratio of P8 results divided by P2 results. A ratio greater than 1 demonstrates an increased sensitivity during the development for LDH and Hoechst data, the opposite for the MTT test. Statistical significance *vs.* DMSO ^a^p < 0.05; ^b^p < 0.01; ^c^p < 0.001. Hip: hippocampus; Cll: cerebellum; IC: inferior colliculus; SC: superior colliculus; Ctx: cortex.

**Table 2:** Analysis of the markers of biomolecular pathway of damage induced by bilirubin.

	Hip	Cll	IC	SC	Ctx
*Ho1*	3.97 ± 1.09^c^	2.55 ± 0.95^b^	3.39 ± 0.92^c^	2.51 ± 0.67^a^	1.65 ± 0.09
*Srnx1*	14.90 ± 2.79^c^	4.38 ± 3.15^a^	17.42 ± 5.65^a^	9.12 ± 4.83^b^	1.44 ± 1.12
*Tnfα*	3.58 ± 2.17^a^	2.06 ± 1.3	1.81 ± 0.94	2.25 ± 0.32^c^	1.08 ± 0.61
*Il1β*	13.69 ± 7.17^b^	8.83 ± 6.08^a^	23.96 ± 10.58^c^	5.38 ± 2.8^c^	2.89 ± 1.51^a^
*Il6*	305.08 ± 120.22^c^	9.64 ± 5.04^c^	98.92 ± 38.35^c^	122.08 ± 126.10^a^	12.39 ± 7.61^c^
*Cox2*	53.64 ± 11.12^c^	62.19 ± 29.95^c^	56.08 ± 21.88^c^	2.48 ± 2.06^a^	3.53 ± 2.44^a^
Glu	2.39 ± 0.31^c^	1.20 ± 0.25	2.03 ± 0.59^b^	1.23 ± 0.18	1.21 ± 0.18

The expression of selected genes, used as markers of biomolecular pathways of damage induced by bilirubin, was expressed as fold change of the control (DMSO-challenged OBCs). Similarly, quantification of glutamate release in the medium of Bf-challenged OBCs was expressed as the fold change of DMSO-exposed cultures. *Ho1*: heme oxygenase1; *Srnx1:* sulfiredoxin 1; *Tnfα*: tumor necrosis factor alfa; *Il1β*: interleukin 1β; *Cox2*: cyclo-oxygenase 2. Data are expressed as the mean ± SD of three-five repetitions. Statistical significance: ^a^p < 0.05; ^b^p < 0.01; ^c^p < 0.001. Hip: hippocampus; Cll: cerebellum; IC: inferior colliculus; SC: superior colliculus; Ctx: cortex.

**Table 3:** Restoration of marker genes levels and glutamate release after cocktail treatment.

	Hip	Cll	IC	SC	Ctx
*Ho1*	164.26^b^	90.20	101.92	110.61	100.94
*Srnx1*	106.6	61.58	51.96	45.65	31.84
*Tnfα*	33.72	91.05	91.46	32.12^c^	62.61
*Il1β*	4.42^b^	27.01	4.26^b^	6.99^b^	20.84
*Il6*	17.67^b^	11.09^b^	7.62^b^	1.16	7.65^b^
*Cox2*	2.60^a^	4.91^b^	2.25^b^	71.99	40.00
Glu	58.53^b^	94.52	66.14^a^	98.04	116.14

The expression of selected genes/glutamate release was expressed as % of gene expression compared with the same sample challenged with bilirubin. *Ho1*: heme oxygenase1; *Srxn1:* sulfiredoxin 1; *Tnfα*: tumor necrosis factor alfa; *Il1β*: interleukin 1β; *Cox2*: cyclo-oxygenase 2; Glu: glutamate. Data are expressed as the means of three-five biological repetitions. Statistical relevance: ^a^p < 0.05; ^b^p < 0.01; ^c^p < 0.001. Hip: hippocampus; Cll: cerebellum; IC: inferior colliculus; SC: superior colliculus; Ctx: cortex.

**Table 4:** Primer specifications.

Gene	Accession number	Forward primer 5′-3′	Reverse primer 3′-5′	Amplicon length (bp)
*Hprt*	NM_012583.2	AGACTGAAGAGCTACTGTAATGAC	GGCTGTACTGCTTGACCAAG	163
*Gapdh*	NM_017008.2	CTCTCTGCTCCTCCCTGTTC	CACCGACCTTCACCATCTTG	87
*Ho1*	NM_012580.2	GGTGATGGCCTCCTTGTA	ATAGACTGGGTTCTGCTTGT	76
*Srxn1*	NM_001047858.3	AAGGCGGTGACTACTACT	TTGGCAGGAATGGTCTCT	85
*Tnfα*	NM_012675.2	CAACTACGATGCTCAGAAACAC	AGACAGCCTGATCCACTCC	172
*IL1β*	NM_031512.2	AACAAGATAGAAGTCAAGA	ATGGTGAAGTCAACTATG	137
*IL6*	NM_012589.1	GCCCACCAGGAACGAAAGTC	ATCCTCTGTGAAGTCTCCTCTCC	161
*Cox2*	NM_017232.3	CTTTCAATGTGCAAGACC	TACTGTAGGGTTAATGTCATC	92

Primer sequences designed for the mRNA quantification. *Hprt* hypoxanthine guanine phosphoribosyltransferase, *Gapdh* glyceraldehyde 3-phosphate dehydrogenase, *Hmox1* Heme oxygenase 1, *Sxrn1* sulfiredoxin 1, *Tnfα* tumor necrosis factor alpha, *Il1β* interleukin 1 beta, *Il6* interleukin 6, *Cox2* cyclooxygenase 2.
